# How can community engagement in health research be strengthened for infectious disease outbreaks in Sub-Saharan Africa? A scoping review of the literature

**DOI:** 10.1186/s12889-021-10348-0

**Published:** 2021-04-01

**Authors:** Samantha Vanderslott, Manya Van Ryneveld, Mark Marchant, Shelley Lees, Sylvie Kwedi Nolna, Vicki Marsh

**Affiliations:** 1 Oxford Vaccine Group & Oxford Martin School, University of Oxford, 34 Broad St, Oxford, OX1 2BD UK; 2 School of Public Health, University of the Western Cape, Robert Sobukwe Road, Bellville, 7535 Republic of South Africa; 3 Department of Global Health and Development, London School of Hygiene & Tropical Medicine, London, UK; 4 Department of Public Health, University of Yaounde I, Rue Melen, Yaounde, Cameroon; 5 KEMRI Wellcome Trust Programme, Nairobi, Kenya; 6 Centre for Tropical Medicine and Global Health, NDM, Oxford University, Oxford, UK; 7 Kenya Medical Research Institute - Wellcome Trust Research Programme, Kilifi, Kenya

**Keywords:** Community engagement, Effectiveness, Health research, Epidemics, Outbreaks, Infectious disease, Sub-Saharan Africa

## Abstract

**Background:**

Community engagement (CE) is a well-established practical and scholarly field, recognised as core to the science and ethics of health research, for which researchers and practitioners have increasingly asked questions about desired standards and evaluation. In infectious disease outbreak contexts, questions may be more complex. However, it is unclear what body of knowledge has been developed for CE specifically as it applies to emerging infectious diseases. This scoping review seeks to describe (1) How CE has been conceptualised and understood; and (2) What conclusions have research teams reached on the effectiveness of CE in these settings, including challenges and facilitators.

**Methods:**

We used a scoping review framework by Arksey and O’Malley (Int J Soc Res Methodol 8:19–32, 2005) to structure our review. We conducted a brainstorming session and initial trial search to inform the protocol, search terms, and strategy. Three researchers discussed, developed and applied agreed screening tools and selection criteria to the final search results. Five researchers used the screening tools to screen abstracts and full text for inclusion by consensus. Additional publications were sought from references of retrieved publications and an expert call for literature. We analysed and reported emerging themes qualitatively.

**Results:**

We included 59 papers from a total of 722 articles derived from our trial and final literature searches, as well as a process of “citation chasing” and an expert call for grey literature. The core material related exclusively to health research trials during the 2014–2016 West Africa Ebola outbreak. We synthesized reports on components of effectiveness of CE to identify and propose three themes as essential elements of effective CE.

**Conclusions:**

While there is a large volume of literature documenting CE activities in infectious disease research settings generally, there are few accounts of effectiveness dimensions of CE. Our review proposes three themes to facilitate the effectiveness of CE initiatives as essential elements of CE activities in infectious diseases studies: (1) Communication towards building collaborative relationships; (2) Producing contextual knowledge; and (3) Learning lessons over time. As there were relatively few in-depth accounts of CE from our literature review, documentation and accounts of CE used in health research should be prioritised.

**Supplementary Information:**

The online version contains supplementary material available at 10.1186/s12889-021-10348-0.

## Background

CE has become an ethical requirement for research involving human participants [52]. Dickert and Sugarman [19] have identified four ethical goals of CE: enhancing protection, enhancing benefits, creating legitimacy, and sharing responsibility. In the 2000s, there were significant developments in CE in clinical trials in Sub-Saharan Africa, especially human immunodeficiency virus (HIV) trials. These changes were motivated by the early closure of pre-exposure prophylaxis (PrEP) trials in Cambodia and Cameroon following protests led by HIV activists who argued trial participants were taking risks but not receiving enough benefits [41]. In response, activists called for a strengthened role of communities in the development and the conduct of HIV trials and pushed for a broader view of CE to promote community empowerment and shared decision-making [50]. Following these calls, there have been a number of successes in promoting dialogue with communities, especially the transformation from an activist-led movement that ‘pushed’ for inclusion, to a researcher-led effort, where study staff worked to encourage participation and ‘pull’ communities into relationships with researchers [38, 50]. Although there have been positive developments in CE, there are concerns that CE in clinical trials does not always address the broader concerns of participants, governments, activists, and researchers themselves, especially political and economic issues related to involving people from resource poor communities [43].

With the growing number of clinical trials around emerging diseases in the last five years, there have been further calls for improved CE, especially in emergency situations. The 2014–16 Ebola Virus Disease (EVD) outbreak in West Africa led to the deaths of more than 11,000 people in Sierra Leone, Liberia and Guinea [57]. Experiences during the response to the epidemic revealed to a broader community of scientists the dangers of ineffective CE, especially mistrust between communities and authorities [27]. In the wake of these experiences, a number of guidelines and reports were published for CE for clinical trials for emerging diseases [15].

The *Good Participatory Practice Guidelines for Trials of Emerging (and Re-emerging) Pathogens (GPP-EP)* [57] set out recommendations for stakeholder engagement that draw from an expert base of actors involved in the EVD outbreak, and borrow from biomedical HIV intervention ‘good participatory practice’ trial guidelines (e.g. [53]). The *GPP-EP* addresses key concepts in stakeholder engagement rather than community engagement, defining stakeholders, ethical issues, and the need for long-term, sustained partnerships. In this document, using the term ‘stakeholder’ rather than ‘community’ in discussing engagement as the focus implies a larger set of health research actors. Here ‘synergy’ between research and response is also seen as crucial to set out ethical principles and ‘optimal practice’ through nine activities throughout the research life-cycle [57]. The *GPP-EP* recommends processes to develop research protocols, budget allocation and time, and collaborative partnering for a “collective shaping of relevant, scientifically rigorous, ethical research that is in line with international standards, respects the rights of the involved population, contributes to and does not undermine the epidemic response, and leaves a sustaining legacy for the involved population” (ibid. p. 4). Effective engagement is both an intrinsic ethical imperative and has instrumental value toward enhancing trial conduct and contributing to robust research outcomes. While the window of opportunity for research during an outbreak is short, trial stakeholder identification and engagement is crucial. The foundational *GPP-EP* principles underpin partnerships with “respect, fairness, integrity, transparency, accountability, and autonomy, while the benchmarks include mutual understanding, complementarity, and efficiency” (ibid. p. 5).

In addition to the World Health Organization (WHO) report, an ad hoc committee was formed at the National Academies of Sciences, Engineering, and Medicine to review and conduct an analysis of the clinical trials conducted during the 2014–2016 EVD outbreak. Their consensus report explores and analyses the scientific and ethical issues related to clinical trial design, conduct, and reporting. The report’s second chapter focuses on “Conducting clinical research during an epidemic” [34]. The core recommendations include: i) begin CE early (recognising contingency of health research and the public health response); ii) ensure that CE is ‘meaningful’ (that is, that experiences are comprehensively and transparently reported and utilised); and iii) maintain sustained funding and investment to develop relationships in inter-epidemic periods. The report recognises the key role of social scientists or anthropologists in learning about cultural, social, political and historical dynamics that could affect CE and research and emphasises promoting the voices of local experts, leaders and community liaison staff. From a practical perspective, the authors describe the need for communication to engage multiple stakeholders in multiple ways, and ensure information is accurately presented, including through translations.

Although these documents provide good guidance on CE, there has been no scoping review of the conduct of CE in emerging disease treatment and prevention trials. At this stage, an assessment of methods for measuring the ‘effectiveness’ of CE would be useful to strengthen and broaden these approaches. However, the existing diversity of definitions and methods for evaluating CE activities may preclude such an effort [2]. For this reason, we have focused this scoping literature review on the practice of CE by aiming to answer two key questions: (1) How has CE been conceptualised and understood? (2) What conclusions have research teams reached on the effectiveness of CE, including the challenges and facilitating factors described?

## Methods

We used a scoping review methodological framework provided by Arksey and O’Malley [4] to structure our review focusing on papers that documented community engagement in health research on infectious diseases. The review stages included: literature search strategy, trial literature search, literature searching, and reference search from final search. We began with a brainstorming session to define the search parameters, including key terms, inclusion and exclusion criteria and appropriate Boolean operators. As some concepts in our questions were ambiguous or difficult to define, we compiled a comprehensive list of search term synonyms.

We then conducted an initial trial search to test our search strategy and make adjustments on one database, Scopus. The trial search used two limits: peer-reviewed papers published after 1990 in English, including all types of research studies (e.g. randomised controlled trials, cohort studies, surveys and qualitative studies). After our trial search we devised our protocol document and registered it on Prospero (CRD42018112501). The 'final' search was conducted across multiple databases.

We also searched through references of publications retrieved from our final search for additional publications. This was a process of ‘backward’ and ‘forward’ citation chasing of included articles [9] for additional follow up references, where we identified linked studies by checking bibliographies of included reviews and conducting citation searches of our core papers. Three researchers (MV, SV, and VM) were responsible for discussing and applying the agreed screening tools and selection criteria to the final search results. Five researchers (MV, SV, MM, VM and SK) used the screening tools to screen abstracts and then full text of selected articles for inclusion by consensus. Finally, we analysed and reported on the emergent themes qualitatively.

An iterative process throughout each of the stages was to clarify key concepts – identifying the key concepts, debating their suitability to the context of health research for infectious disease outbreaks in Sub-Saharan Africa and producing a final list with related definitions. We concentrated on the accounts of CE in six clinical trials for which a substantial account of these activities is given. Our final list is outlined in detail in the next section.

### Key concepts

In this review, ‘health research’ is taken to include all health-related biomedical research. ‘Emerging (and re-emerging) infectious disease outbreaks’ are those for which there are few or no countermeasures to control the transmission [56]. These outbreaks may also be referred to as epidemics if they spread across geographic areas.

CE – ‘Community engagement’ – is a more ambiguous term that encompasses a number of assumptions. A central assumption in the reference to the ‘community’ is that there are uniform communities and that these are readily identifiable. Marsh et al. [38] acknowledge that there are challenges such as defining ‘community’ and addressing power differentials between researchers and communities. As Wilkinson et al. [58] describe, ‘community’ can also be a socio-political construct underpinned by complex hierarchies and politics. The form of ‘community’ often differs across different types of health research. For example, in treatment trials, participants are recruited from an (often narrow) patient base, while in vaccine trials participants may come from a broader community of residents in a given geographic area. There is also a lack of clarity between CE being an ethical requirement with intrinsic value (mutual respect) and practical requirement with instrumental value (making health research feasible, acceptable, and maximising benefits).

We refer to ‘engagement’ to include the time in anticipation of (before), during and after outbreaks (including future considerations) and across all forms of ‘engagement’ or interaction. At the same time, we recognise that ‘engagement’ is also a contested term and encompasses many activities with varying goals [9]. Sherry Arnstein’s ‘Ladder of participation’ [5] is a popular representation of the different levels of citizen involvement from a situation of manipulation of citizens through categories of non-participation, to tokenism, to citizen power, towards an open society of cooperation. The form of engagement is often contingent on research methods, though this is rarely explicit [37]. A highly visible, large-scale clinical trial may necessitate more robust CE that puts at least some community members or trial participants in decision making roles alongside researchers. This type of engagement might be said to democratise a research endeavour that might otherwise risk being coercive or invasive. This legitimisation of research institutions and practices is no less important during epidemics, but it is likely to be more challenging operationally for two main reasons. First, many institutions’ relationships and practices are more fluid during outbreaks, and therefore less predictable and more difficult to operate in. Second, any CE for research in outbreaks is likely to be conducted alongside engagement for the public health response.

Good practice in CE that achieves its aims is often described in terms of ‘effectiveness’, though there are other terms used (e.g. strength, quality, and impact) [17]. To assess whether CE has been effective, the goals of engagement need to be clear. In practice, CE activities can have multiple aims that are sometimes conflicting, and approaches to measuring achievement of these will be similarly complex [42]. Given the contestations around aims of CE and what constitutes good practice, we employ the ‘broad tent’ World Health Organization (WHO) definition: “a process of developing relationships that enable stakeholders to work together to address health-related issues and promote well-being to achieve positive health impact and outcomes” ([59], p. 12).

### Critical appraisal, coding and synthesis under themes

We used three guidelines for quality appraisal. These guidelines were the ‘Critical Appraisal Skills Programme’ (CASP) systematic review checklist [16], the ‘Confidence in the Evidence from Reviews of Qualitative research’ (CERqual) approach [36] to determine robustness of results and the Authority, Accuracy, Coverage, Objectivity, Date, Significance (AACODS) checklist for critical evaluation of the grey literature [35].

We draw on our findings to offer an analysis of major elements of CE that are central to ‘effectiveness’ or good practice in research through the development of three core themes. We arrived at the themes during the analysis phase, during which we sorted all the sources into categories beginning with disciplinary approaches and general topic areas to be refined and reordered into themes based on the objectives and outcomes of CE. During this iterative process, we discussed the contents of each paper, condensing what we had assessed and through these discussions derived themes, which were continually refined. See Additional file 1 for the underlying conceptual framework of the three core themes for effective CE.

## Results

The total number of papers identified through database searching was 460. In addition, the trial search returned 203 papers, which were screened for relevance based on abstracts and titles (there were no duplicates as this search was only run on one database). We found 17 articles relevant to our question. After examining the overall relevance of our trial search results and consulting a librarian and experts in this field, we adapted search terms slightly before moving onto the final search. However, 14 'missing' articles from the initial trial search, which we had deemed as relevant to our questions based on their title or abstract but which did not appear in the final search due to the changes in search terms, were included (the remaining three articles that we had deemed relevant did appear in the new search and thus were not included as these would have been duplicates). The total number after duplicate articles were removed was 470.

We screened these 470 titles and abstracts and excluded 412, leading us to a total of 58 articles for full text screening. Five people were involved in the full text screening of 58 articles, at which point a further 12 articles were excluded. In total the articles excluded on title and abstract screening were 412. The full text screening was conducted using a standardised screening template with the characteristics outlined above, for which we also noted whether the paper was included and any follow-up references. From the full text screening, we generated a list of 50 follow-up references, through ‘citation chasing’. We also made a call for literature at the African coaLition for Epidemic Research, Response and Training (ALERRT) consortium annual general meeting held in March 2019 in Dakar, Senegal, based largely on recommendations received by collaborators from the consortium as well as pieces encountered during searching and screening. This resulted in 9 pieces of grey literature being collected from the recommendations of experts.

Of this additional literature (grey and follow up references) a total of 46 articles were excluded. The main reason for excluding grey literature and citation chasing sources was the content not being substantive enough. MV, SV and VM met regularly to discuss screening choices and approaches to synthesis and analysis. At the final stage a total of 59 articles were included for analysis in the review.

### Accounts of CE in the literature

In the following section, we describe the CE activities undertaken in six clinical trials, what was described as ‘working’ or not, why, and any lessons suggested. We then describe the scope covered by a wider relevant literature on CE, including research accounts that reference CE activities and commentaries and analyses of what constitutes good practice for CE. Drawing on our findings, we offer an analysis of major elements of CE that are central to ‘effectiveness’ or good practice in research. We then go on to present three themes that summarise the conceptualisation and understandings of CE and the conclusions research teams reached on the effectiveness of CE in their settings.

Our final search across the electronic databases EBSCOhost, OVID, Proquest, Pubmed, Scopus and Web of Science returned 460 results. (See Additional file 2. for full search terms.) After deduplication using the reference software Mendeley, this was reduced to 267. Two of the authors (MV and SV, with oversight from VM) conducted a title and abstract screening round, which left articles marked as ‘yes’ (include), ‘flagged for interest’ or ‘unsure’. There were also 14 non-duplicated references from our initial trial search that we had flagged as relevant but did not come up in our main search due to changes in search terms. These were added manually to our spreadsheet of references for full text screening. We then extracted data using the characteristics in Table 1 below.

**Table 1:** Characteristics for data extraction

Context descriptors	
Aims/objectives	
Research type/topic	
Community organisers	
Community organising actions and mechanisms of engagement	
Tools/medium	
Community target/definition	
Views on effectiveness/strengthening/quality/impact	
Experience, success and lessons/learnings	
Exiting	

**Fig. 1:**
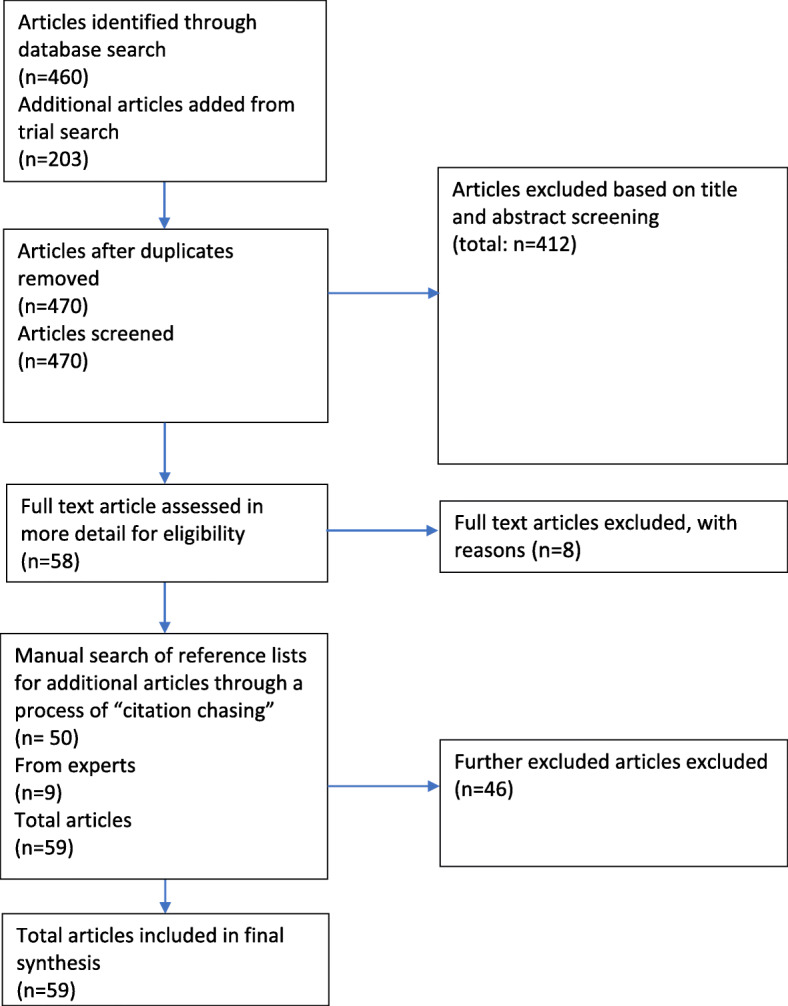
PRISMA diagram

The Reporting Items for Systematic Reviews and Meta-Analyses (PRISMA) diagram in Fig. 1 above outlines our methodological process (see Additional file 3 for PRISMA Checklist).

#### Empirical research (trials) with substantial CE accounts

Papers on CE activities for Ebola treatment and prevention trials in Sierra Leone, Liberia and Guinea were the most substantial accounts of CE. These were either vaccine trials (four), which mostly recruited healthy volunteers from a wide community base, or treatment trials (two), which recruited in-patients during their stay in isolation facilities. The vaccine trials aimed to recruit healthy volunteers and had the strongest focus on CE as part of their recruitment strategy and associated need to engage with a wide area of local residents.

Table 2 below summarises the set-up, aims, and mechanisms of the CE strategy and activities, as reported in the literature linked to six trials conducted during the West Africa Ebola outbreak between 2014 and 2016.^1^ The trials varied in nature, size, design and in the scope of their reporting on CE aspects of their research. While all of the trials described CE to some degree – usually in relation to ethical principles such as those used in Good Clinical Research Practice – they used a variety of justifications, approaches and priorities as described later in this section. The difficulty in describing detail is that, from our review, it seems that CE for clinical trials is either (i) not generally written up as a research activity (for example, to evaluate progress towards goals) but as a process, or (ii) write-ups are not comprehensive, particularly where published papers are primarily reporting on trial activities and outcomes. For our analysis, therefore, we have gathered what is relevant and available from the literature.

**Table 2:** Trial name, intervention, targeted recruitment population, reported CE strategy, activities, and outcomes

Trial Name	Intervention	Targeted recruitment population	Reported CE strategy	Reported CE activities and outcomes
	**EBOLA VACCINE TRIALS** [20, 34]			
STRIVE (completed)	Vaccine trial phase III; individually randomised, open-label trial; immediate vs deferred vaccination; 8673 participants enrolled.	Non-pregnant, frontline EVD health workers or related care workers across 5 districts in Sierra Leone.	Dedicated significant time and resources for CE. This meant communication beginning before trial launch (and presentation to the full government/ media). Communication continued post-enrolment to support ongoing recruitment and participation.	Communication activities included a 24-hour hotline for questions. Educational activities involved more than 175 sensitisation and information sessions for potential participants, hospitals, community health centres and ETUs.
Ebola ça Suffit (completed)	Ring vaccination; novel cluster RCT; immediate vs deferred vaccination; 7284 participants enrolled.	Index cases and their contacts within an epidemiologically informed ring.	Social mobilisation began prior to any vaccination related activities taking place. Consent was gained from the main ring site where the vaccination took place, around the index patient’s residence.	‘Social mobilisation experts’ to find cases and contacts who they sought to mobilise and gain consent for participation. Community leaders and representatives assisted in contacting patients where applicable. The experts explained the trial’s objectives and implications of potential participation.
Ebovac Salone (ongoing)	Vaccine trial Phase III (staged).	Healthy volunteers, Sierra Leone.	Iterative CE approach strongly informed by prior and ongoing qualitative research. ‘Research-driven community engagement’ seen as contributing to smooth recruitment and reducing disruption due to rumours and misinformation.	Dedicated social science team and community liaison teams aimed to understand intra-community power dynamics. Conclusion that local understandings of fairness can inform the recruitment strategy design and rumours can be addressed through ‘active dialogue’ rather than on correcting misinformation. This emerging understanding was used to support and adapt CE over the course of the trial.
PREVAIL (completed)	Vaccine trial phase II/III; randomised, double-blind, placebo-controlled trial; 1500 participants enrolled.	Liberian residents aged > 18 years. High risk communities proximal and distal to an identified referral hospital in Monrovia.	Social mobilisation strategy with four pillars: advocacy, communications, community engagement and monitoring and evaluation.	Activities included: Reaching out to community decision-makers, opinion leaders and political leaders for support and approval, targeted messaging, answering FAQs, print and broadcast media communication, distributing flyers, jingles and songs on television and radio, text message communications with telecommunication companies’ subscribers etc.
	**EBOLA TREATMENT TRIALS** [47]			
JIKI (completed)	Treatment trial Phase II; non-comparative, single-arm, open-label clinical trial.	Any patient aged > 1 year with lab confirmed EVD; four rural Ebola treatment centers (ETCs) in Guinea.	The trial organisers recognised the context of fear and mistrust of international actors. The main recruitment efforts were inside the four ETCs involved in the study. A CAB was set up and involved in discussions on trial protocol, CE approach and informed consent processes.	A pretrial initiative was used to inform and involve community leaders, and develop ‘thoughtful, culturally appropriate messages’ and a consensual community strategy. The trial was conducted in partnership with public health response NGOs.
Ebola Tx. (completed)	Treatment trial, phase II/III. Open-label, nonrandomised clinical trial.	Donor mobilisation of EVD survivors. Lab-confirmed EVD patients	Strong focus on donor mobilisation and role of Survivors Association-motivation to donate linked to feelings of social responsibility as survivors.	Issues identified were the stigma and perceptions of health impacts of donating blood – a decrease in vital strength and antibodies, fears of loss of acquired protection against EVD.

#### Other literature on CE during outbreaks: commentaries on and shorter references to CE

In addition to reports of CE embedded in trials conducted during the 2014–16 EVD outbreak, across the trial-related literature there is a strong recognition of the importance of CE in facilitating the implementation of health research. For example, Folayan et al. [30] identify four critical stakeholders and associated tasks that need to be implemented before clinical trials begin. These are: (1) Global research coordinating body (WHO); (2) Affected governments; (3) Ethics committees; and (4) Community Advisory Boards (CABs). The associated tasks centred around data-sharing and exploiting synergies, access and leverage of resources, reviewing/monitoring for ethical integrity, and working with ethics committees. Processes that clarified the roles and expectations of implementing partners for better CE included the use of Standard Operating Procedures (SOPs) [46]. The use of SOPs was to provide national and district-level guidance, such as recruiting and training CE practitioners and how to structure social mobilisation or CE leadership alongside the rest of the response effort.

In this review, the value of CE is often framed in terms of encouraging support for the trial and the language used to describe this tends to be linked to promotion, enhancement and sensitisation [33, 47]. For example, the instrumental value of CE is recognised for overcoming challenges to both the outbreak research and the public health response, in addressing rumours and changing core risk behaviours, such as those around ‘unsafe’ burial practices [10] and in introducing new measures like contact tracing and quarantine [45].

In addition, one published report summarises the vaccination campaigns that were a major part of health research during the 2014–2016 EVD outbreak in West Africa. The Wellcome Trust and Center for Infectious Disease Research and Policy (CIDRAP) report “Recommendations for Accelerating the Development of Ebola Vaccines” [14] provides a review and analysis of the various aspects of research and development for Ebola vaccines, to provide an expert framework in the “global efforts to accelerate the availability of effective and safe Ebola vaccines” (ibid. p. 1). The report mostly focused on post-marketing Ebola vaccination campaigns, rather than trials or other health research activities. Despite the post-marketing framing, it still offers general CE recommendations that can be applied to health research. Consideration of early strategies for CE for vaccine deployment is included as well as descriptions of previous experiences where CE played an important role in the successful rollout of vaccines.^2^ The treatment trials were not reported on as a group in this Wellcome and CIDRAP report. An example of the type of research conducted included testing the effectiveness of giving EVD survivor plasma to patients, where the CE activities included outreach to EVD survivors to request plasma donation.

All of these trials received ethical approval from the relevant international and national stakeholders [3, 39], so they have demonstrated an intention and plan to conduct research that complies with ethical principles in relation to informed consent, favourable risk-benefit ratio, social value, respect for persons and communities [22]. The diversity with which these principles are interpreted and enacted in individual studies is clearly complicated by the extreme challenges faced when conducting health research in an outbreak setting.

In the following sections, we describe three interrelated themes that emerged from our analysis as capturing activities as essential elements that constitute ‘effective’ CE. The three interrelated themes are: (1) Communication towards building collaborative relationships; (2) Producing contextual knowledge; and (3) Learning lessons over time. In addition to interplay between these themes, there is also variation from the influence of other elements of the research, including trial design, human resources, and logistics management. This interpretive framework helps us to paint a picture of the on-the-ground experience, lifting out nuance and details that might otherwise be lost.

#### Three CE themes

##### Theme 1: communication towards building collaborative relationships

The need for accurate and culturally sensitive communication with relevant community stakeholders about a trial is widely recognised as a critical preliminary step in the research process [11, 51]. As is well described in the literature, communication can serve a range of purposes, from more ‘one-way’ or outreach forms of communication to more ‘bi-directional’ or participatory forms-supporting learning and consultation, for example.

In addition, as for CE as a whole, some purposes in communicating may have more intrinsic than instrumental value, although these may be difficult to tease apart. An example of an intrinsic value (or ‘good in itself’) would be communication that realises respect for individuals or communities. Reynolds and Sariola call this intrinsic value the “moral ideals of scientific research” ([48], p. 257). This is relevant to the role of listening to, understanding and responding to a variety of conceptualisations of disease and health responses to it, which are often peppered with mistrust, fear and confusion in the context of an infectious disease outbreak. Communication then has instrumental value in its ability to clarify messages, tackle mistrust and generate support for research, but is also intrinsic in allowing the lived experiences of stakeholders to be respected, heard, recognised and integrated into the functioning of the research.

Unpacking the explicit and implicit aims of communication methods — particularly in the context of setting up a new trial, perhaps in a research-naïve community — can lay the groundwork for continued communication strategies that are cognisant of their multiple effects. While broadcasted communication can be useful in getting information to a large audience, there are challenges in using this approach to spread ‘correct’ messages or address rumours and misinformation if it is not accompanied by multi-directional, bottom up, interpersonal forms of communication, allowing for sources, justifications and ontologies of these rumours and misinformation to emerge and be understood [24, 28].

A helpful interpretation of this general approach to communication in the context of outbreak research is given by Kennedy et al. who reframe ‘one-way’ communication as a more instrumental process, for example around the ‘dispelling’ of myths and misconceptions ([33], p. 52); and ‘bi-directional’ forms as more intrinsic, revealing concerns about health interventions “stemming from histories of mistrust, rather than simply being misunderstandings” ([26], p. 8). Instrumental aims might include increasing recruitment alongside the dispelling of myths (perhaps also to improve recruitment and retention) but falls short of calls in the research ethics literature for collaborative partnerships with community, health provider and policy stakeholders (at national and local levels) to be a basic building block of ethical practice [21].

Trial reports make it clear that both one-way (outreach) and bi-directional (collaborative) communication strategies were used in practice. One core and seemingly obvious reason for paying more attention to ‘outreach’ is the number and distribution of potential participants in a trial. The three trials in Sierra Leone and Liberia that involved large numbers of healthy volunteers across wide geographic areas (Ebola ca Suffit, Ebovac Salone, and PREVAIL) paid more attention to outreach than others, and also built in bi-directional communication processes. In contrast, the trials recruiting volunteers from health workers (STRIVE) and patient or survivor populations (JIKI and Ebola Tx) largely reported on interpersonal communication strategies, at patient/patient group and policy stakeholder levels, including — in the case of Ebola Tx — an association of survivors to secure plasma donors needed for the intervention. Throughout the accounts in this section, differences in the ‘community’ to be engaged related to the nature of the trial and was reflected in chosen approaches.

Reflecting the variation in CE approaches with that of trial design, all teams recognised the importance of a wide range of communication activities, from outreach to bidirectional and interpersonal. For example, the PREVAIL team described using multiple communication strategies in different ways to achieve “an exceptionally high participant retention rate (97%)”, despite numerous logistical and operational challenges ([8], p. 50). Throughout the following sections, we provide examples of different communication activities and the authors’ reflections on these in terms of ‘effectiveness’ or satisfying aims.

#### Broad outreach activities

Across the vaccine trials in the core literature, communication was regularly enacted as forms of outreach or ‘broadcast’, for example through “the local media, regular press conferences and distribution of flyers, preparation and airing of jingles and songs on television and radio, and distribution of text messages” ([33], p. 52). One large communication campaign was the ‘Ebola Big Idea of the Week’ campaign run by the CDC. It involved approximately 80 radio, television, and print journalists from Sierra Leone trained by experts to develop one critical idea every week through culturally sensitive messaging [6]. For PREVAIL, the strategic plan for ‘social mobilisation’ included outreach elements of branding, print and broadcast media communication through distributing flyers, television, radio and mobile phone messaging [8]. The aim of these mechanisms was getting ‘facts’ to a wide audience in order to clarify, support and inform through a largely one-way process, but also to be part of a wider approach to targeting myths, rumour and suspicions.

Both of the treatment trials included in the core literature describe a strong focus on CE with patients and government health authorities responsible for public health programming, including service delivery and epidemic control measures in the surrounding communities. The Ebola Tx treatment trial, which involved transfusion of plasma from EBV donors, had a particularly strong focus on engagement with the EBV Survivors Association, whose cooperation and support underpinned feasibility of the intervention. Reports from the JIKI treatment trial in Guinea gave particularly clear accounts of the challenges and processes for implementation of the trial at field level. At the first site, a treatment centre in Guéckédou, Perez et al. [47] describe that CE was understood as “essential to ensuring the appropriate implementation and progression of the trial” (ibid. p. 7). The challenges faced for CE included the mistrust of governments and Western humanitarian agencies, and rumours about EVD. In response, communication about the trial objectives and implementation was seen as needing messages that “were adapted to context, minimised fear, and managed inappropriate expectations”. A central element of this CE response was the setting up of a Community Advisory Board (CAB), comprising local leaders, women’s organisations, youth groups, religious leaders and village elders (ibid. p. 22). However, the success of instituting a CAB is difficult to assess from the research articles. Other closely related mechanisms to CABs were also used. For example, the Ebovac Salone research team also set up a Participant Advisory Group (PAG) in the initial stages of their formative research and their CE strategy involved a wide range of community stakeholders “from elected and traditional leaders to individual households” ([25], p. 4). CE was designed in this respect to link with on-the-ground organisations and proved valuable for implementing research in an emergency ([44], p. 4). However, CABs are potentially more reflective of collaborative partnerships than some other forms of communication and engagement given the positions of authority that community members have on them. The PAG may appear more limited in scope but may provide meaningful participant advisory in a limited time period of a trial.

#### Interpersonal and key stakeholder communication

All trials included elements of communication targeting individuals or specific groupings of potential participants or ‘influencers’. For the vaccine trials, a study involving health workers in Sierra Leone used a designated hot line and information sharing meetings for potential participants and the wider health workforce. The PREVAIL team highlight interpersonal, local, face-to-face communication as a critical strategy. One such strategy was employing 25 Liberians living in the main town in the trial area as ‘participant trackers’ to engage in ongoing conversations about daily experiences of being in the trial. The feedback about these ‘participant trackers’ was that crucial information was collected in determining the names of participants (in the communities some residents were only known by nicknames), their locations (using descriptions if there were no street names and relying on family and friends to have knowledge of their whereabouts), and the ability to contact them (e.g. a phone number of a community store) [8]. While Browne et al. do not comment on the potential for these kinds of strategies to be intrusive or coercive, they do see ‘effectiveness’ in the high participant retention, and the success of strategies such as home visits in avoiding any overt opposition and dissatisfaction. Another interpersonal communication strategy reported was supporting members of the clinical team (nurses and clinical monitors) to take on communication roles for the trial as part of their everyday interactions with participants and others, by reinforcing messages and tackling stigma, myths and rumours ([8], p. 52).

As a community-based vaccine trial, EBOVAC-Salone set up a dedicated Community Liaison team that worked closely alongside trial researchers and a social science team throughout the study. The main role of the Community Liaison team was to manage community messaging and communication, and conditions for participation and informed consent, through community and household-level meetings alongside community leaders (Fayia [29], p. 37).

The literature emphasizes how closely the trial, community liaison and social science teams worked together, aiming to inform “research-driven communication strategies” ([24], p. 5). For example, they describe their process of holding weekly meetings where findings and feedback could be reported, synthesised and acted upon. This approach (which overlaps with an aspect of CE discussed later – incorporating findings and feedback loops) allows for a communication strategy that is not only instrumentally effective in managing rumours and myths and sharing accurate information but creates a platform for daily experiences to be voiced and incorporated into the trial’s work. Mooney et al. cite an example where a rumour that the vaccine trial was bringing Ebola back to a community was identified by social scientists and responded to by the community liaison team, who visited the local marketplace known to be the place where the rumour had originated. They were then able to engage with concerned citizens directly, “with the support of pre-briefed local stakeholders” ([44], p. 439).

The team at STRIVE (a Phase III vaccine trial involving front line health workers in Sierra Leone) also highlight communication as important in building community trust and mitigating rumours and misinformation [11]. The main rationale described for their engagement was to encourage recruitment, protect participants, and get buy-in at higher levels of national leadership [12]. The communication team, with the help of social mobilisation experts (SMEs) [20], were responsible for ensuring voluntary, informed participation [11]. The approach to participation was based on a tailored social ecology model “to identify and reach specific spheres that influence a potential participant’s decision-making” (ibid. S. 41). This model is a way of showing how an issue can be influenced at multiple societal levels and was used to reach people from different spheres with different norms, influences, perceptions, and support, as well as to foster understanding particular to each sphere (ibid. S. 41). In this trial, informal communication was used instrumentally, where investing in communication training for all staff was part of creating a facilitating environment for conversations seen as at least as constructive as the formal communication efforts (ibid. S. 46).

##### Theme 2: producing contextual knowledge – formative social science research

Formative research is “the process by which researchers or public health practitioners define a community of interest, determine how to access that community, and describe the attributes of the community that are relevant to a specific public health issue” ([13], p. 2). The use of formative research prior to and for the purpose of informing trial design and implementation is a core part of CE strategy. The experiences of trials conducted during the West Africa EVD outbreak demonstrate this. The work done within the EBOVAC-Salone trial is particularly illustrative of this, as described by Enria et al. [24] as the “real-time social science research”. A locally-recruited social science research team operated alongside a locally-recruited community liaison team, and overall clinical research team, with formative social science research playing a crucial role [26]. The social science team included four research assistants from Kambia District where the trial was located, a data analyst and a transcriber, with supervision from an LSHTM social scientist (ibid.). They used a range of traditional qualitative research methods, including interviews and ethnographic observation in local social areas and trial clinics in the three months prior to the trial clinical work. This ‘stage 1’ research and a second support ‘stage 2’ continued alongside the clinical part of the trial. A critical aspect of the EBOVAC-Salone team’s use of social science research is that it went past the formative phase and continued throughout the trial’s CE process.

In terms of the types of social science methods employed, for the Ebola treatment trial with convalescent plasma, an anthropological pre-trial assessment was carried out to better understand the context, acceptability of EVD therapies, stakeholders expectations ([18], p. 648) as well as volunteer motivations, concerns, and their underlying influences [49]. It is unclear who conducted the assessment or whether results were published as a separate set of findings; however, the findings demonstrated the importance of stakeholder communication “to understand and follow up what people thought, felt, perceived, and how they acted during the EVD outbreak and consequential health control activities” ([18], p. 648). This led to discussions about how stakeholders, particularly members of the survivors association that was set up, could be involved in the study. Another example of a reflection of social science methods is the systematic literature review by Johnson and Vindrola-Padros, “Rapid qualitative research methods during complex health emergencies” [32] and subsequent rapid review methodology articles relating to COVID-19 pandemic research [54, 55]. Rapid qualitative methods were effective in understanding community resilience for public health emergencies and planning CE based on this.

Other social science research concentrated on specific aspects of community perceptions and behaviours that affected the public health response. This included motivations for volunteering in research studies (Fayia [29]); the reception to the “bushmeat ban” and public health messaging about the risks of consuming bushmeat [7]; to wider questions about the connection to understandings of citizenship and belonging through encounters with biomedicine [23].

A number of questions arise in the use of formative research to understand context during epidemics for future consideration. Can anthropological research be done in such a short period, especially in a research-naïve area? Who are the anthropologists doing this research? What if the results of their research suggest an unsuitable setting for clinical research? Could the entire trial be relocated given the extreme logistical and time constraints? In other words, what are the specific intentions and aims of the formative research - to prime the setting or to ask open-ended questions about the ethical, social, cultural and historical feasibility of conducting trials in the setting? These studies have shown that these broader questions have largely gone unanswered and will need to be considered for future formative research in outbreaks.

##### Theme 3: learning lessons over time – incorporating findings, creating feedback loops and building a sustaining legacy

To learn lessons means relying on collaborative partnerships so that the various stakeholder ideas and recommendations are taken seriously and built into plans going forwards. The feedback of this information often takes place outside formal structures such as CABs. Knowing and understanding whose feedback is getting incorporated and why is an important step in mapping out the effectiveness of CE strategy. Browne, et al. [8] note how PREVAIL nurses responded to participants’ experiences of stigma by reiterating messages relayed by the Social Mobilisation Experts (SMEs) as part of their broader strategy. Community health workers (CHWs) are also highlighted by Miller et al. [40] as playing an important role during the EVD outbreak and remained active in their communities after. This multiplicity of roles and the use of different stakeholder channels for reinforcement of messaging is an on-the-ground reality difficult to capture in descriptions of formal communication structures and strategies.

The PREVAIL trial’s fourth social mobilisation strategy pillar was monitoring and evaluating mobilisation activities, including “joint review mechanisms that incorporated community leaders, diverse community groups, and government partners to ensure output, impact and challenges of the social mobilisation campaign were identified and changes instituted address existing gaps” ([33], p. 53). The trial design was adapted as a result of incorporating feedback. Study protocols are fairly inflexible once finalised, but in cases where it is possible, getting feedback on the study design before it is finalised (usually through formative research) from local stakeholders and community members is essential for including communities in the design of research.

The EBOVAC-Salone recruitment strategy was strongly informed by clinical expertise, conversations with local staff and insights from anthropological research, and thus shaped according to both clinical standards and local perceptions of what a “fair” and “representative” selection process should be: “Given assumptions that access to resources is assumed to be based on “connectocracy”, there was the potential that, given the limited number of participants required, people could have assumed that the “big ones” were picking themselves and those they knew” ([24], p. 6). Sierra Leone’s history of conflict opened the potential for “participant recruitment to be likened to forced conscription” and added sensitivities for the trial recruitment process (ibid.). Table 3 below outlines the stages of feedback loops.

**Table 3:** Stages of feedback loops. Based on article by [24]: p. 4

1. CE plans feedback	Social science team feedback to study team on CE plans based on socio-cultural research, local community dynamics and perceptions of the vaccine trial.
2. Meetings to discuss issues	Brought up issues encountered by the trial team/ community liaison staff requiring further research by social science team (e.g. design of the recruitment strategies).
3. Reporting rumours/ concerns	The social science team reported on rumours or concerns and communicated these to the community liaison team anonymously (not to breach confidentiality and to maintain independence of research).
4. Response by community liaison staff	Following feedback, the community liaison staff brainstormed strategies to respond to concerns and rumours. Strategies depended on specific issues raised, but usually involved different creative avenues for discussion with community and reviewing messaging to actively engage, as well as determining who was the best person in the team to respond and through which channel.

Compensation and plausible benefit was provided, such as the EBOVAC-Salone trial offering participants compensation for transportation to the clinic but other trials at different locations in the country at that time offered more money and sometimes a mobile phone ([44], p. 439). Some saw the provisions of incentives as a worrying commodification tantamount to “exchanging blood, one’s life essence, for money” and generous financial compensation was not necessary to stimulate participation in the trial in Kambia (ibid.). Still, the Liberian trial team assigned dollar amounts appropriate for the PREVAIL trial setting with compensation for inconvenience, which may have contributed to retention: A baseline visit was $40.00, follow-up blood draw was $20.00, and close out visit was $150.00 ([8], p. 53). Building future infrastructure was another way for CE to have both intrinsic and instrumental value. For example, the formation of the ‘Global Emerging Pathogens Consortium’ (GET), a civil society group comprised of African academics, scientists, clinician, and civil society as a crisis response network, is an example of an enduring legacy for CE [1]. 

We have summarised our findings through three themes: (1) Communication towards building collaborative relationships; (2) Producing contextual knowledge; and (3) Learning lessons over time. See Table 4 below for the full list of all papers in this review, divided specifically by the themes and also whether they were general trial or trial-related papers.

**Table 4:** Literature divided by trials and three themes

Ebola trials and trial-related	1. Communication Towards Building Collaborative Relationships	2. Producing contextual knowledge: Formative social science research	3. Learning lessons over time: Incorporating findings, creating feedback loops and building a sustaining legacy
Abramowitz et al. 2018 [2] Alirol et al. 2017 [3] Caleo et al. 2018 [10] Coltart et al. 2017 [15] Dean et al. 2016 [17] Emanuel et al. 2004 Folayan et al. 2015 [30] Keusch et al, 2017 [34] Marsh et al, 2008 [38] Marchant & Lees, 2019 [37] Mehand et al. 2018 [39] Mills et al. 2005 [41] Olu et al. 2016 [45] Pedi et al. 2017 [46] Slevin et al. 2008 [50] Tindana, 2007 [52] UNAIDS, AVAC. 2011 [53] Wellcome Trust and CIDRAP, 2015 [14] WHO, 2017 [59] WHO, 2016 [57] Wilkinson et al. 2017 [58]	Bedrosian et al. 2016 [6] Browne et al. 2018 [8] Callis et al. 2018 [11] Carter et al. 2018 [12] Ebola ça suffit consortium 2015 [20] Emanuel et al. 2005 [21] Enria et al. 2016 [24] Enria et al. 2016a [25] Enria et al. 2016b [26] Fairhead 2016 [28] Perez et al. 2017 [47] Fayia Tengbeh et al. 2018 Fayia [29] Kennedy et al. 2016 [33] Mooney et al. 2018 [44] Reynolds & Sariola 2018 [48] Spengler et al. 2016 [51]	CDC. 2013 [13] Bonwitt et al. 2018 [7] Delamou et al. 2016 [18] Enria and Lees 2018 [23] Enria et al. 2016 [24] Fayia Tengbeh et al. 2018 Fayia [29] Ronse et al. 2018 [49] Johnson & Vinndrola-Padros, 2017 [32]	Abayomi et al. 2016 [1] Browne, et al. 2018 [8] Callis et al. 2018 [11] Delamou et al. 2016 [18] Enria et al. 2016 [24] Henao-Restrepo et al. 2016 [31] Kennedy et al. 2016 [33] Miller et al. 2018 [40] Mooney et al. 2018 [44]

Across our review of 59 papers, the core material related exclusively to health research trials during the 2014–2016 West Africa Ebola outbreak in Liberia, Sierra Leone, and Guinea. In addition, even though we included studies from 1990, the majority were from 2015 onward. In some cases, reports of CE practices were limited to a few paragraphs in articles primarily focused on other elements such as study design and reporting results [18, 31, 33]. However, some trial groups authored full-length publications in which community engagement aspects were amongst the main foci of the paper [11, 44].

##### Discussion: Key elements for ‘effectiveness’ in CE

We have reviewed the literature on CE for health research for infectious disease outbreaks in Sub-Saharan Africa. While there is a large volume of literature documenting CE activities in infectious disease research settings generally, there are few accounts of effectiveness dimensions of CE. Our review proposes the consideration of three themes by researchers to facilitate the effectiveness of their CE initiatives. After identifying the high-level principles and benchmark documents we looked to the three core areas of literature of ethics, public health response, and outbreak research; then we assessed how these translated to on-the-ground CE.

In the previous sections, we have described three themes that emerged from our analysis as particularly important in informing the effectiveness of CE planned and implemented during health research in outbreaks. Clearly, research design has a crucial influence on the nature of CE planned, and in particular, the extent to which geographically defined communities might be implicated by the research activity.

We have made a first core argument that communication approaches should be multifaceted and tailored to context, but include elements that build collaborative relationships between partners. A helpful interpretation of this general approach to communication in the context of outbreak research is given by Kennedy [33] who reframes ‘one-way’ communication as a more *reactive* process, for example around the dispelling of myths and misconceptions ([33], p. 52), and ‘bi-directional’ forms as more *exploratory*, revealing and taking seriously the concerns about health interventions held by communities where the research is situated [26].

The move from ‘one-way’ forms of communication towards bi-directional communication or even collaborative relationships implies a shift from less to more participatory forms of CE, as has been commented on elsewhere in this article. Unpacking the different implications of these two (sometimes conflicting) approaches to CE is important for clarifying and achieving its aims. Whether CE is undertaken for exploratory or reactive reasons, the question of who benefits is still relevant, as is that of how fairly the benefits are distributed. Reluctance to overburden the public health response team features strongly among researchers and practitioners designing and implementing CE for research in outbreaks, noting that CE for a public health response and in support of research may rely on the same teams. Overcoming impediments of mistrust, conflicting perceptions, and misunderstandings of the relationship between research and response are also acknowledged in the literature on CE for research in outbreaks.

We have made a second argument that social science research-formative and ongoing-is a critical component in guiding the shape and content of CE during an outbreak and ensuring that the collaborative relationships researchers aim to build are well-founded, meaningful and durable. Our third argument then stems from the first two, to highlight the importance of a commitment to learning lessons from on-going experiences during an outbreak, so that research teams can flexibly adapt and sustain the collaborative relationships on which the research enterprise depends. We extend this third argument further, to agree with the authors of the WHO *GPP-EP* guidelines that in view of long-standing structural inequities that characterise settings in which outbreaks are likely to arise, there should be a long-term commitment to supporting a sustainable legacy for communities.

Our review suggests that good practice in CE in the context of outbreak research (and potentially for research in general) is underpinned by bi-directional communication, the careful longitudinal incorporation of social science research into planning, and ensuring that structures are in place to support meaningful feedback and adaptation of research practice on the ground. This includes efforts to ensure that CE has an enduring legacy from research, for the benefit of a widely defined community. Through this scoping review we have found that, while CE research literature documents the activities of CE conducted in the context of clinical research during disease outbreaks, the next step of deriving the essential elements of meaningful and effective engagement has not been taken and is what makes this scoping review novel. Going forward, the additional work needed in order to further knowledge in assessments of CE will be to consider broader implications of CE on an aggregate level, taking into consideration the kinds of research – largely qualitative – that may answer some of these questions.

However, we do acknowledge the following limitations. First, we made the decision to limit our research to Sub-Saharan Africa. A comparative global review may prove useful, particularly with the Americas and Asian regions where different outbreaks have taken place recently (Dengue, Zika etc.), inviting varied CE responses. In light of the COVID-19 pandemic, a truly global review for CE responses would also be very valuable. Second, our review was largely confined to the English language, and only reviewed one paper in French. Further language searches would add to the body of knowledge.

## Conclusion

We have presented our findings from a synthesis of three key themes arising from descriptions of CE strategies and their effectiveness, as essential elements of CE activities in infectious diseases studies: (1) Communication towards building collaborative relationships; (2) Producing contextual knowledge; and (3) Learning lessons over time. Researchers and their partners implementing CE programs for health research in epidemics can use these themes to guide improving practice.

Throughout the process of conducting this review (and also confirmed at the ALERRT 2019 Community Engagement Workshop in Senegal), it has become apparent that strengthening CE is both an art and a process, a philosophy and a science. Achieving both the instrumental aims (effectiveness, strengthening, quality, and impact) and intrinsic aims (respect, recognition, and equality) of CE requires a deliberative effort through the nexus of relationships, knowledge, and lesson-learning for better listening to and speaking with communities affected by outbreaks of infectious diseases. The importance of documenting and sharing lessons learnt to improve CE practice remains paramount as current epidemics continue.

We set out to characterise the nature and effectiveness of CE activities in infectious disease research settings in Sub-Saharan Africa. Given the nature of the wider literature on community engagement in research, our findings act to underline their interrelatedness of these themes and their core role in supporting ‘effectiveness’ across the literature reviewed on CE during the EBV epidemic. There is value in concentrating on these areas in planning CE in similar situations in future. As there were relatively few in-depth accounts of CE from our literature review, documentation and accounts of CE used in health research should be prioritised going forward, thus contributing to more thorough trial reporting in the future. A further lesson is the need for more research and analysis on what constitutes effectiveness in CE in research, and greater transparency in reporting around the processes and impacts of CE. A deeper understanding through more comprehensive reporting of the mechanisms of CE would provide stakeholders with a benchmark for assessing the ethical principles around CE and offer a tangible, on-the-ground opportunity to enact them.

## Supplementary Information

**Additional file 1.** Conceptual framework of 3 core themes for effective CE. We show a diagrammatic representation of the nature of and interplay between these three elements, illustrating the way they may work together towards supporting effective CE and providing a ‘marker’ for community responsiveness. Relevant benchmarks from the ‘state-of-the-art’ reports are shown in the diagram, indicating their role in an overall analysis

**Additional file 2.** Scoping Review Search terms. Search terms of trial search on Scopus and final search across all included databases

**Additional file 3.** PRISMA Checklist. Preferred Reporting Items for Systematic reviews and Meta-Analyses extension for Scoping Reviews (PRISMA-ScR) Checklist

## Data Availability

No additional data are available. The Review Protocol is published online at Prospero: Registration number CRD42018112501 and available at https://www.crd.york.ac.uk/PROSPERO

## Abbreviations

ALERRT: African coaLition for Epidemic Research, Response and Training
CAB: Community advisory board
CERqual: Confidence in the Evidence from Reviews of Qualitative research
CASP: Critical Appraisal Skills Programme
AACODS: Authority, Accuracy, Coverage, Objectivity, Date, Significance
CDC: Centers for Disease Control and Prevention
CE: Community engagement
CHW: Community health workers
CIDRAP: Center for Infectious Disease Research and Policy
ETC: Ebola treatment center
EVD: Ebola virus disease
HIV/AIDs: Human immunodeficiency virus/acquired immunodeficiency syndrome
LMI: Low- and middle-income countries
MNCH: Maternal, newborn, and child health services
PRISMA: Reporting Items for Systematic Reviews and Meta-Analyses
RCT: Randomised controlled trial
SME: Social mobilisation expert
WHO: World Health Organization

## Footnotes

1 One of these trials is still ongoing (the Ebovac Salone trial). The literature included in this section represents the results of our search and may not include very recently published articles even if related to these trials. The dominance of the 2014–2016 West Africa EVD outbreak is a striking characteristic of our overall search results, particularly given that we made an effort to keep our search terms as inclusive of all the major WHO blueprint diseases as possible. This suggests that there are significant gaps in the literature regarding CE for health research in other infectious disease outbreaks.

2 Reversing resistance to polio vaccination in northern Nigeria, random selection for clinical trials of Malaria vaccines in Burkina Faso by using traditional games, and frequent, widespread and multi-layered communication strategies for the Meningitis Vaccine Project in Central Africa (ibid. p. 40–41).
