# Supplementary material for: How can community engagement in health research be strengthened for infectious disease outbreaks in Sub-Saharan Africa? A scoping review of the literature
Source: BMC Public Health. 2021 Apr 1;21:633. doi: 10.1186/s12889-021-10348-0 (PMC8012744; doi:10.1186/s12889-021-10348-0)
Supplement: Supplementary file 1 — Additional file 1. Conceptual framework of 3 core themes for effective CE. We show a diagrammatic representation of the nature of and interplay between these three elements, illustrating the way they may work together towards supporting effective CE and providing a ‘marker’ for community responsiveness. Relevant benchmarks from the ‘state-of-the-art’ reports are shown in the diagram, indicating their role in an overall analysis [file 12889_2021_10348_MOESM1_ESM.docx]

## Appendix 1. Conceptual framework of 3 core themes for effective CE

We show a diagrammatic representation of the nature of and interplay between these three themes, illustrating the way they may work together towards supporting effective CE and providing a ‘marker’ for community responsiveness. Relevant benchmarks from the ‘state-of-the-art’ reports are shown in the diagram, indicating their role in an overall analysis.

*Limit*:

Exploitation, trial-related harm, one-size-fits-all, cross-cultural miscues, misinformation

*Promote*:

Follow-up/results/dissemination, post-trial access to products, sustained funding/investment

- Incorporating findings
- Creating feedback loops
- Sustaining legacy, preparedness & capacity-building

Reflexivity

- Multiple stakeholder identification, collaboration & partnership
- Communication, information presentation & giving voice
- Capacity building & fostering leadership
- Facilitation through community advisory boards/ liasions
- Formative social science research
- Role of social scientists/ anthropologists
- Informing & supporting implementation

*Constraints*: Timing, budgets, vaccine delivery uncertainty

*Benefits*: Early action, experiences comprehensively, transparently, trust,

inclusivity, matching strategies to countries

*Processes:*

Planning, issues management, protocol development, policies, standards

**Figure 1.** Proposed conceptual framework of 3 core elements for effective CE.

The corresponding table below provides an overview for all of the key concepts in the state-of-the-art reports.

| Type  Principles [Values] & Ethics | Overarching view of ‘meaningful’ CE to enhance trial & contribute to outcomes, recognising health research & public health response are contingent. Need for responsibility and sharing.  Underpinning principles of collaboration (respect, fairness, integrity, transparency, accountability & autonomy)  Benchmarks (mutual understanding, complementarity & efficiency)  International ethical standards (respect rights, contribute to epidemic response, have a sustaining legacy)  Other guiding ethical principles: social value, scientific validity, fair selection of study population, favourable risk-benefit ratio, independent review, informed consent, & respect for recruited participants/ study communities. |
| --- | --- |

**Table 3. Types of CE, principles, and ethics**

| Type  Components  Processes  [practicalities/  contribute to]  Promote  Prevent  Limits  Principles [Values]  Ethics | CE enhance trial & contribute to outcomes, recognising health research & public health response are contingent  Partnerships/collaboration, capacity-building, formative research, role of social scientists/ anthropologists communication, stakeholder identification, multiple stakeholders, voice to locals  Planning, issues management, protocol development, policies, standard-setting  Benefits, early action, prevention/care, follow-up, results, dissemination, post-trial access to products/procedures, sustained funding/investment, experiences comprehensively/transparently report/utilised, trust, West African leadership, inclusivity, lessons learned, match strategies to countries  Exploitation, trial-related harm, one-size-fits-all, cross-cultural miscues, misinformation  Timing, budgets, vaccine capability delivery uncertainty  Responsibility, respect, sharing, ethical, underpinning principles (respect, fairness, integrity, transparency, accountability & autonomy), benchmarks (mutual understanding, complementarity & efficiency), ‘meaningful’ CE  International standards - Informed consent, respect rights, contribute to epidemic response, sustaining legacy |
| --- | --- |

| Emanuel et al. 2004. “What Makes Clinical Research in Developing Countries Ethical? The Benchmarks of Ethical Research” | |
| --- | --- |
| (1) **Partnerships**  (2) **Responsibility** (planning, conducting and overseeing research, disseminating results and integrating into health-care system  (3) **Respect** community’s values, cultures, traditions, social practices  (4) **Capacity-building** for stakeholders to become full & equal partners  (5) Participants and communities receive **benefits** from research  (6) **Share** fairly research rewards | - ‘Collaborative **partnership’** to reduce risk of **exploitation** in health research (with poverty, limited health-care infrastructure, low research literacy) - Other guiding **ethical** principles (social value, scientific validity, fair selection of study population, favourable risk-benefit ratio, independent review, informed consent, respect for recruited participants/study communities) |
| The WHO *GPP-EP* guidelines (2016) | |
| (1) **Formative** research activities  (2) **Stakeholder engagement** **plan**  (3) **Communications/** **issues** management plan  (4) **Protocol** development  (5) **Informed consent** process  (6) Standard of **prevention and care**  (7) Policies on trial-related **harms**  (8) Trial accrual, **follow-up, and exit**  (9) Trial **closure, results, dissemination, post -trial access** to trial products/ procedures. | - Develop research **protocols** to budget allocation/ time - Collaborative **partnering** for collective shaping of relevant/scientifically rigorous/ethical research - International **standards** (respect rights, contributes to epidemic response, sustaining legacy) - Both **ethical** imperative & instrumental value to **enhance trial**/ contribute to outcomes - Trial **stakeholder identification/** engagement - Underpinning **principles**: respect, fairness, integrity, transparency, accountability & autonomy, benchmarks inc. mutual understanding, complementarity & efficiency |
| USA National Academies Report (2017) | |
| (1) Begin **CE early** (recognising health research & public health response are contingent)  (2) **‘Meaningful’ CE** & ensure experiences comprehensively/transparently report/utilised  (3) **Sustained funding & investment** to develop resources in inter-epidemic periods | - Key role of **social scientists/ anthropologists** to learn about cultural, social, political & historical dynamics - **Voice to local** experts/leaders/ community liaison staff - **Communications** to engage multiple stakeholders & accurately present information |
| Wellcome Trust and CIDRAP report “Recommendations for Accelerating the Development of Ebola Vaccines” (2015) | |
| Challenges   1. **Timing** 2. A **one-size-fits-all** approach does not exist for West Africa 3. Lack of **trust** 4. **Misinformation** 5. Uncertainties about vaccine delivery **capabilities** 6. **Cross-cultural** miscues | Recommendations   1. Begin immediately 2. Promote West African leadership 3. Promote inclusivity and collaboration 4. Employ lessons learned to inform Ebola strategies 5. Match strategies to each country 6. Ensure **transparency** |
