# Supplementary material for: How can community engagement in health research be strengthened for infectious disease outbreaks in Sub-Saharan Africa? A scoping review of the literature
Source: BMC Public Health. 2021 Apr 1;21:633. doi: 10.1186/s12889-021-10348-0 (PMC8012744; doi:10.1186/s12889-021-10348-0)
Supplement: Supplementary file 2 — Additional file 2. Scoping Review Search terms. Search terms of trial search on Scopus and final search across all included databases [file 12889_2021_10348_MOESM2_ESM.docx]

**Appendix 2.**

**Scoping Review Search terms**

Search terms of trial search on Scopus and final search across all included databases.

**Search terms of trial search on Scopus:**

TITLE-ABS-KEY ( "community engagement" OR "stakeholder engagement" OR "social mobilisation" OR "community capacity" OR "behaviour change" OR "participat*” OR "communication" OR "community mobilisation" OR "sensitisation" OR "health promotion" OR "disease prevention" OR "public engagement" OR "community led" OR "village" OR "village led" OR "community driven" OR “community based” OR "grassroots" OR "awareness" OR "consultation" AND research OR trial AND epidemic OR outbreak OR "complex health emergency" AND africa AND effectiv* OR “best practice” OR “good practice”)

**FINAL SEARCH ACROSS ALL INCLUDED DATABASES**

"community engagement" OR "stakeholder engagement" OR "social mobilisation" OR "social mobilization" OR "community capacity" OR "behavi* change" OR participation OR communication OR "community mobilisation" OR "community mobilization" OR sensitization OR sensitisation OR "health promotion" OR "disease prevention" OR "public engagement" OR "community led" OR "village" OR "community driven" OR grassroots OR awareness OR consultation* AND "Africa South of the Sahara" OR angola* OR benin* OR "Burkina Faso" OR burundi* OR "Central African Republic" OR chad* OR congo* OR “Democratic Republic of Congo” OR “DRC” OR "Cote D'Ivoire" OR djibouti* OR ethiopia* OR eritrea* OR gabon* OR gambia* OR ghana* OR guinea* OR kenya* OR lesotho* OR liberia* OR malawi* OR mali* OR mauritania* OR mozambique* OR namibia* OR niger* OR rwanda* OR senegal* OR "Sierra Leone*" OR somalia* OR "South Africa*" OR sudan* OR swaziland* OR tanzania* OR togo* OR uganda* OR "United Republic of Cameroon" OR zaire* OR zambia* OR zimbabwe* AND "research" OR trial* OR intervention AND "Communicable diseases" OR outbreak* OR "hemorrhagic fever" OR "crimean hemorrhagic fever" OR ebola OR "lassa fever" OR "marburg virus" OR "rift valley fever" OR "MERS-CoV" OR "Middle Eastern Respiratory Syndrome cornovirus" OR sars OR "Severe Acute Respiratory Syndrome" AND effectiv* OR "best practice" OR "good practice"
